# Nicotine as Therapy

**DOI:** 10.1371/journal.pbio.0020404

**Published:** 2004-11-16

**Authors:** Tabitha M Powledge

## Abstract

In daily use for centuries by hundreds of millions of people, nicotine has only lately been investigated for its therapeutic potential in a long list of common ills

There's a cheap, common, and mostly safe drug, in daily use for centuries by hundreds of millions of people, that only lately has been investigated for its therapeutic potential for a long list of common ills. The list includes Alzheimer disease, Parkinson disease, depression and anxiety, schizophrenia, attention deficit hyperactivity disorder (ADHD), and even pain and obesity. Why has interest in this potential cure-all been slow to develop? One reason: in its current forms the drug offers pharmaceutical companies no possibility of substantial profit. Another, perhaps more important: the drug is reviled as the world's most addictive. The drug, of course, is nicotine.

Nicotine is an alkaloid in the tobacco plant Nicotiana tabacum, which was smoked or chewed in the Americas for thousands of years before European invaders also succumbed to its pleasures and shipped it back to the Old World. Nicotine has always been regarded as medicinal and enjoyable at its usual low doses. Native Americans chewed tobacco to treat intestinal symptoms, and in 1560, Jean Nicot de Villemain sent tobacco seeds to the French court, claiming tobacco had medicinal properties and describing it as a panacea for many ailments. Higher doses are toxic, even lethal—which is why nicotine is used around the world as an insecticide. Yet few of the horrendous health effects of smoking are traceable to nicotine itself—cigarettes contain nearly 4,000 other compounds that play a role. Until recently, nicotine research has been driven primarily by nicotine's unparalleled power to keep people smoking, rather than its potential therapeutic uses.

Nicotine locks on to one group of receptors that are normally targeted by the neurotransmitter acetylcholine. Nicotinic acetylcholine receptors (nAChRs) are ion channels threaded through cell membranes. When activated, either by acetylcholine or by nicotine, they allow selected ions to flow across the cell membrane. In vertebrates nAChRs are all over the autonomic and central nervous sytems and the neuromuscular junction. A nAChR is composed of five polypeptide subunits (Figure 1), but there are many nAChR subtypes made of different subunit combinations, a diversity that helps explain why nicotine can have so many different physiological and cognitive effects.

**Figure 1:**
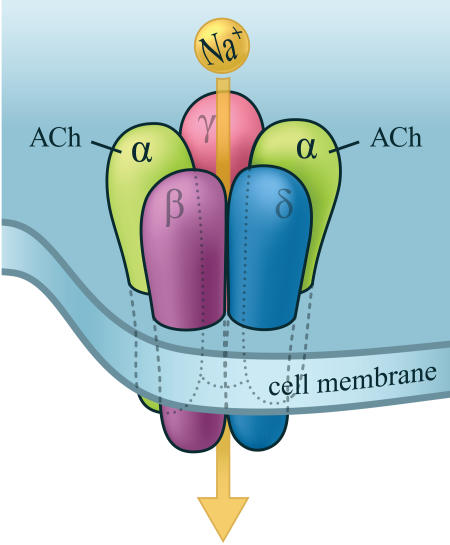
Schematic Illustration of an Acetylcholine Receptor (Illustration: Giovanni Maki)

It is now conventional wisdom that acetylcholine and nicotine act at these receptors to alter electrochemical properties at a variety of synapses, which can in turn affect the release of several other neurotransmitters. This wisdom exists thanks in part to work by Lorna Role and her colleagues at Columbia University in New York City. “In 1995, we turned people's attention to how nicotine works as a modulator, tuning synapses and increasing the gain on transmitter release,” Role recalls. Although all nAChRs are activated by nicotine, other drugs could be found or designed that affect only a subset of these receptor types. “If you can dissect out the important players with respect to which nicotine receptors are tuning [a] particular set of synapses, then that provides another way to potentially target the therapeutics.”

## Nicotine and the Brain

People with depressive-spectrum disorders, schizophrenia, and adult ADHD tend to smoke heavily, which suggested to researchers that nicotine may soothe their symptoms. Common to all these disorders is a failure of attention, an inability to concentrate on particular stimuli and screen out the rest. Nicotine helps. Researchers at the National Institute on Drug Abuse have shown via functional magnetic resonance imaging that nicotine activates specific brain areas during tasks that demand attention (Box 1). This may be because of its effects, shared with many other addictive drugs, on the release of the neurotransmitter dopamine. “Schizophrenia is a disorder largely of the dopamine system,” says John Dani of the Baylor College of Medicine in Houston, Texas. Dopamine signals in the brain occur in two modes—a kind of background trickle, punctuated by brief bursts. “It's thought that schizophrenics have a hard time separating that background information from important bursts. We've shown that nicotine helps to normalize that signaling by depressing the background but letting the bursts through well,” he says. “I'll be surprised if there's not a co-therapy [to help schizophrenics] that takes advantage of nicotine systems in less than a decade.”

**Box 1. Nicotine's Effect on Attention**

**Figure 2:**
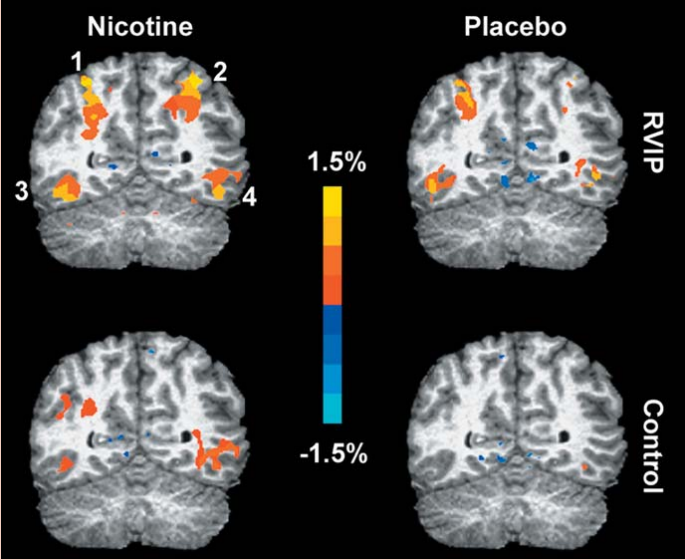
The Brain on Nicotine (Image: Elliot Stein, National Institute on Drug Abuse)

Using functional magnetic resonance imaging, scientists at the National Institute on Drug Abuse provided the first evidence that nicotine-induced enhancement of parietal cortex activation is associated with improved attention. They compared brain activity during a task demanding sustained attention—rapid visual information processing (RVIP)—with that during an undemanding sensorimotor control task (Figure 2). Group results from 15 smokers (right) illustrate the effects of nicotine and placebo patches in left and right parietal cortex (1 and 2) and left and right occipital cortex (3 and 4). Nicotine significantly increased activation in occipital cortex during both the control and rapid visual information processing tasks, suggesting a general modulation of attention. In contrast, nicotine increased activity in the parietal cortex only during rapid visual information processing, suggesting a specific modulation on task performance.

Nicotine may be the link between two genes that appear to figure in schizophrenia. Sherry Leonard and Robert Freedman of the University of Colorado in Denver, Colorado, have shown that expression of the gene for the alpha 7 neuronal nicotinic receptor is reduced in schizophrenics, and have argued that alpha 7 abnormalities lead to attention problems. Researchers in Iceland and elsewhere have shown that a different gene, for the growth factor neuregulin, also appears to figure in the disease. Neuregulin, Role and her colleagues have shown, governs the expression of nAChRs in neurons and helps to stabilize the synapses where they are found. The researchers are currently studying interactions between neuregulin and alpha 7, which Role thinks will prove important.

Smokers also have lower rates of neurodegenerative disorders, and nicotine improves cognitive and motor functioning in people with Alzheimer disease and Parkinson disease. The prevailing hypothesis is that nicotine increases release of neurotransmitters depleted in those diseases. Dani and his colleagues have recently shown that acetylcholinesterase inhibitors—which block the degradation of acetylcholine and hence prolong its action—used to treat Alzheimer disease also stimulate dopamine release. They suspect that malfunctioning of the dopamine system may be affecting noncognitive aspects of dementia such as depressed mood, and that this might be alleviated by nicotine.

Paul Newhouse and his colleagues at the University of Vermont in Burlington, Vermont, are studying nicotine drugs as potential therapeutic agents for cognitive dysfunction. Newhouse, a long-time nicotine researcher, is heading the first study ever to examine the efficacy and safety of nicotine patches for treating mild cognitive impairment, thought to be a precursor of Alzheimer disease. The researchers hope to see a positive effect on attention and learning. Newhouse also heads two studies of nicotinic stimulation in ADHD, using the patch, nicotine blockers, and some novel drugs that activate nicotine receptors.

## Nicotine and Pain

Nicotine's salutary effects in patients with neurodegenerative and mental disorders have been studied a lot and are fairly well known. Two much newer topics of academic research are nicotine's potential for pain relief and for treating obesity.Nicotine itself has provided modest pain relief in animal studies. Although the analgesic effect of drugs that mimic acetylcholine were originally attributed to a different class of receptors, it is now clear that nAChRs play an important role in the control of pain. For instance, epibatidine, a drug that is extracted from the skin of an Ecuadorian frog and that acts at nAChRs, has been shown to be 200 times more potent than morphine at blocking pain in animals. Current animal research is aimed at discovering just where, how, and which classes of nAChRs work against pain, with the aim of developing more selective drugs.

Meanwhile, nicotine is also being investigated as an analgesic in humans. For example, Pamela Flood, an anesthesiologist at Columbia, is investigating nicotine's future as a postoperative analgesic. She recently completed a pilot study of 20 women undergoing gynecological surgery. All the women had access to unlimited morphine and also got either a single 3-mg dose of nicotine nasal spray or a placebo. The placebo group had peak pain scores of eight out of a possible ten in the first hour after surgery. Women who got nicotine averaged a pain score of five. Despite the small sample size, Flood says, the results were highly significant. “As far as I know this is the first clinical study to use nicotine for analgesia, and it was much more successful than I ever would have imagined.”

“The nice thing about nicotine and drugs like nicotine is that they have opposite side effects to anesthetics. Instead of being respiratory depressants, they are respiratory stimulants. Instead of being sedating, they increase alertness. So theoretically this class of drugs is actually the perfect thing to add to an opioid regimen. The fact that they're synergistic was a fortuitous thing that we had never looked at, and neither had anybody else.”

## Nicotine and Weight Gain

Nicotine may be the most effective drug around for weight control. As ex-smokers know, to their rue, one of the worst things about quitting cigarettes is putting on pounds—as much as 10% of body weight. “Something about being addicted to nicotine and then going off it causes massive increase in weight,” Role points out.

Young-Hwan Jo in Role's lab is looking at a particular brain circuit involved in motivational behavior, especially feeding behavior. It is lodged primarily in the lateral hypothalamus but has projections all over the cortex, especially the nucleus accumbens, which is the center of reinforcement. “This is where information that has come in to the thalamus and the hypothalamus is relayed to cortical areas with some sense of salience or remembrance. It presumably is involved in changing perception and motivation for eating. It's not, ‘I have to eat this,’ it's, ‘I want to eat this,’” says Role.

Jo has been comparing the synaptic effects of nicotine, which reduces appetite, to those of cannabinoids, which stimulate it. “Control of these projection neurons seems to be oppositely regulated by these two,” Role notes. “It doesn't necessarily mean we've found the root of the munchies, but it at least points to pathways that these things have in common.” Jo is also examining how nicotine and cannabinoids modulate these pathways in genetically obese mice, and also their interactions with leptins. Role says tuning these pathways up or down might be a reasonable aim. “If that could be done in a selective fashion, maybe that could be introduced in appetite control. Certainly I see…antagonism of some of these pathways that nicotine activates or the complementary activation of the cannabinoid pathways as very important targets for therapeutics with respect to the anorexia that's associated with chemotherapy.”

Ming Li and his colleagues at the University of Texas in San Antonio, Texas, are studying nicotine's effects on weight and on expression of genes that nicotine upregulates orexin and neuropeptide Y and, more recently, that it also regulates leptin signaling. All three molecules regulate feeding behavior controlled by the hypothalamus. In the weight study, nicotine-treated rats not only lost weight, they lost about 20% of their body fat compared to saline-treated controls. The researchers suggest that, among its other effects, nicotine alters fat storage.

**Figure 3:**
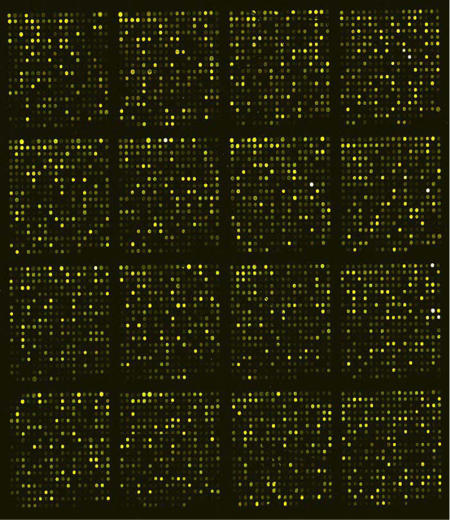
Microarray Showing Patterns of Gene Expression Influenced by Nicotine (Image: Ming Li, University of Texas Health Science Center at San Antonio)

The University of Texas researchers have scoured the literature for genes related to nicotine, and they are developing microarrays to study the expression of these genes (Figure 3). While nicotine seems to affect all the molecules known to influence weight, Li says it's clear the story is even more complex. “That's the reason we keep looking at different molecules, to find key targets involved in this regulation.” The ultimate hope is to develop new drug applications.

Dani predicts that weight control is likely to be one of the earliest nicotine-based therapies. “There's a very good chance that the first drug is unlikely to be…nicotine itself, but will take advantage of nicotinic receptors in the therapy,” he says. “I know there are drugs now being tested by drug companies just for that purpose.”

## Nicotine's Future

Developing new drugs that selectively target specific subtypes of nicotine receptors is an expensive, albeit potentially lucrative, proposition. And therein lies a question. Will nicotine-based therapy consist mostly of costly new drugs from the pharmaceutical industry? Or can less expensive nicotine products like the patch, chewing gum, and nasal spray—which are generally intended for smoking cessation but widely available, usually without prescription—find their way into the world's medicine cabinets?

“It's a little early to call whether nicotine will be used itself as a therapeutic agent or whether these more specific drugs that are being produced or maybe even used in combination with other drugs may be the most important way to go,” says Dani. But he doesn't see the medicinal use of plain nicotine as very likely. Dani points out that the body's own agent, acetylcholine, acts over milliseconds to activate nicotinic receptors, whereas nicotine itself stimulates these receptors for hours. That lengthy action means that, although nicotine activates the receptors, it then often turns particular receptor subtypes off again, a process called desensitization. “It's hard to predict inside of a body what you're getting. Am I getting an activation or am I turning the receptors off?”

Yet much of the work to date showing nicotine's effectiveness on a huge range of disorders has involved products available at any drugstore and intended to help people quit smoking. Newhouse is using patches for mild cognitive impairment. Flood has demonstrated pain relief with nasal spray and will use patches in her next study. And Role feels that gum hasn't been adequately explored for its therapeutic potential. Nicotine gum, she notes, is a better imitator of smoking than the patch because it delivers brief hits rather than a steady supply. She's also uncertain whether natural nicotine has been studied enough. But Role also points out that nicotine has its serious problems—addictive potential, cardiovascular damage, and (especially when delivered through the mucosa) cancer.

Dani says, “People are probably going to have to find creative ways to understand which subtypes of nicotinic receptors they're turning on and which ones they're desensitizing. Maybe drug delivery methods will matter. Maybe subtype specificity will matter. It's less than a decade that we've known how important nicotinic receptors are. Now we have to move forward from there.”

“We've made an enormous amount of progress on understanding the biology of these receptor systems and how to target them. What has been trickier has been to develop an appropriate pharmacology that allows one to selectively target agents for particular therapeutic purposes with an adequate safety index,” Newhouse says. “But some of the drugs that are coming on in human trials now are very promising. So I'm cautiously optimistic that we're on the road to developing some useful nicotinic therapies.”

## Abbreviations

ADHD: attention deficit hyperactivity disorder
nAChR: nicotinic acetylcholine receptor

## References

[pbio-0020404-Flood1] Flood P, Sonner JM, Gong D, Coates KM (2002). Isoflurane hyperalgesia is modulated by nicotinic inhibition. Anesthesiology.

[pbio-0020404-Freedman1] Freedman R, Adams CE, Adler LE, Bickford PC, Gault J (2000). Inhibitory neurophysiological deficit as a phenotype for genetic investigation of schizophrenia. Am J Med Genet.

[pbio-0020404-Li1] Li MD, Kane JK (2003). Effect of nicotine on the expression of leptin and forebrain leptin receptors in the rat. Brain Res.

[pbio-0020404-McGehee1] McGehee DS, Heath MJ, Gelber S, Devay P, Role LW (1995). Nicotine enhancement of fast excitatory synaptic transmission in CNS by presynaptic receptors. Science.

[pbio-0020404-Newhouse1] Newhouse PA, Potter A, Singh A (2004). Effects of nicotinic stimulation on cognitive performance. Curr Opin Pharmacol.

[pbio-0020404-Yang1] Yang X, Kuo Y, Devay P, Yu C, Role L (1998). A cysteine-rich isoform of neuregulin controls the level of expression of neuronal nicotinic receptor channels during synaptogenesis. Neuron.

[pbio-0020404-Zhang1] Zhang L, Zhou FM, Dani JA (2004). Cholinergic drugs for Alzheimer's disease enhance in vitro dopamine release. Mol Pharmacol.

